# Comparative Genomics and Functional Studies of Wheat BED-NLR Loci

**DOI:** 10.3390/genes11121406

**Published:** 2020-11-26

**Authors:** Clemence Marchal, 10+ Wheat Genome Project, Georg Haberer, Manuel Spannagl, Cristobal Uauy

**Affiliations:** 1 John Innes Centre, Norwich Research Park, Norwich NR4 7UH, UK; clemence.marchal@jic.ac.uk; 2 Plant Genome and Systems Biology, Helmholtz Center Munich, D-85764 Neuherberg, Germany; georg.haberer@helmholtz-muenchen.de (G.H.); manuel.spannagl@helmholtz-muenchen.de (M.S.)

**Keywords:** NLR, integrated domain, NLR-ID, BED-NLR, plant disease resistance, zf-BED, wheat, Yr5, Yr7

## Abstract

Nucleotide-binding leucine-rich-repeat (LRR) receptors (NLRs) with non-canonical integrated domains (NLR-IDs) are widespread in plant genomes. Zinc-finger BED (named after the *Drosophila* proteins Boundary Element-Associated Factor and DNA Replication-related Element binding Factor, named BED hereafter) are among the most frequently found IDs. Five BED-NLRs conferring resistance against bacterial and fungal pathogens have been characterized. However, it is unknown whether BED-NLRs function in a manner similar to other NLR-IDs. Here, we used chromosome-level assemblies of wheat to explore the *Yr7* and *Yr5a* genomic regions and show that, unlike known NLR-ID loci, there is no evidence for a NLR-partner in their vicinity. Using neighbor-network analyses, we observed that BED domains from BED-NLRs share more similarities with BED domains from single-BED proteins and from BED-containing proteins harboring domains that are conserved in transposases. We identified a nuclear localization signal (NLS) in Yr7, Yr5, and the other characterized BED-NLRs. We thus propose that this is a feature of BED-NLRs that confer resistance to plant pathogens. We show that the NLS was functional in truncated versions of the Yr7 protein when expressed in *N. benthamiana*. We did not observe cell-death upon the overexpression of Yr7 full-length, truncated, and ‘MHD’ variants in *N. benthamiana*. This suggests that either this system is not suitable to study BED-NLR signaling or that BED-NLRs require additional components to trigger cell death. These results define novel future directions to further understand the role of BED domains in BED-NLR mediated resistance.

## 1. Introduction

Plants have evolved a wide diversity of immune receptors to perceive biotic stresses and trigger defense responses [1]. Among these receptors, nucleotide-binding leucine-rich-repeat (LRR) receptors (NLRs) constitute a large family of intracellular sensors of pathogen-associated molecules. To prevent the spread of infection, NLRs trigger a series of signaling steps ultimately leading to cell death upon pathogen recognition [2]. The conserved domain organization in NLR proteins includes a nucleotide binding NB-ARC domain and leucine-rich repeats (LRRs) at the C-terminal of the protein. Three broad classes of NLRs are defined based on the nature of the conserved domain located at the N-terminal of the protein: CC-NLRs carry a coiled-coil (CC) domain, TIR-NLRs a Toll and Interleukin-1 Receptor (TIR) domain, and RWP8-NLRs a RPW8-like-CC domain. These N-terminal domains are likely involved in the activation of defense response [3].

Within these major classes, a sub-category of NLRs carry additional non-canonical domain(s), typically referred to as integrated domains (NLR-IDs). The position of these integrated domains varies within the protein [4] and functional studies have confirmed their role in direct recognition of pathogen-associated molecules (e.g., effectors) [5,6,7,8], leading to the definition of the ‘integrated decoy model’ [9]. This model proposes that NLR-IDs have evolved from the integration of the effector target in the canonical NLR structure. A recent study also showed that IDs could also indirectly recognize effectors via direct interaction with the effector target, suggesting that some NLR-IDs ‘guard’ the effector target [10].

Several in silico analyses have shown that NLR-IDs are found in numerous plant genomes and that protein kinases, WRKY, and zinc-finger BED (named BED hereafter) correspond to some of the most frequently identified integrated domains [4,11,12]. BED-NLRs (i.e., an NLR with an integrated BED domain) were found in plant species belonging to the Poaceae, Fabaceae, Salicaceae, and Myrtaceae families [4,12] and are preferentially located at the N-terminus of the NLR [4,13]. Although the function of the BED domain within BED-NLRs is unknown, this protein architecture is functional against both bacterial and fungal pathogens [14,15,16,17,18]. For example, in barley, *Rph15* encodes a BED-NLR conferring resistance against *Puccinia hordei*, the causal agent of barley leaf rust [17]. In rice, both *Xa1* and *Xo1* encode BED-NLR proteins that confer resistance against *Xanthomonas oryzae* pv. *oryzae* (*Xoo*) and *Xanthomonas oryzae* pv. *oryzicola* (*Xoc*), the causal agents of bacterial blight (BB) and bacterial leaf streak, respectively [14,15,16]. For *Xa1*, it was hypothesized that the BED domain was an integrated decoy that could act as an effector trap for TAL-effectors that target specific promotor sequences in the host plant [19]. Both *Xa1* and *Xo1* possess a nuclear localization signal (NLS) upstream of their BED domains, and the N-terminus of *Xo1* fused to GFP localizes in nucleus upon overexpression in *Nicotiana benthamiana* [16]. 

Little is known about the general function of the BED domain in plant proteins, apart from its ability to bind DNA and its presence in hAT transposases [20,21]. There is evidence for a family of BED domain-containing proteins, the *SLEEPER* proteins, to act as transcription factors in plants [22,23]. Knock-out mutants of certain *SLEEPER* genes in rice and Arabidopsis have detrimental developmental effects [22]. However, whether the function of BED domain from BED-NLRs are related to BED domains found in these particular proteins remains to be determined. 

Previously, we cloned three BED-NLRs (*Yr5, Yr7, YrSP*) conferring resistance to *Puccinia striiformis* f. sp *tritici* (*Pst*), the causal agent of yellow rust of wheat [18]. While *Yr7* is closely related to *Yr5*, we found that *Yr5* is allelic to *YrSP*. Hence, these two genes are now referred to as *Yr5a* and *Yr5b* (Robert McIntosh, personal communication). The BED domains of *Yr5a* and *Yr5b* are identical, yet these two alleles confer different resistance spectra to *Pst* [18]. This is similar to what was observed in *Xa1* and *Xo1*, which carry identical BED domains and yet recognize different pathogens [16]. This suggests that, in this case at least, the BED domain does not solely govern pathogen recognition specificity. It is unclear whether Yr7 and Yr5a/b proteins encode a coiled-coil domain given that predictions vary depending on the program used [17,18]. We also investigated the degree of similarity between BED domains from all wheat proteins (BED-NLRs and non-NLR proteins) with a neighbor-network analyses. We found that BED domains were highly diverse and that only a handful of BED domains from non-NLRs proteins shared similarities with BED domains from BED-NLRs [18]. This is consistent with the hypothesis that integrated domains may have evolved to strengthen the interaction with pathogen effectors after integration [24]. It is unclear, however, if this holds true across a wider dataset and if the BED domains found in NLRs act as an ‘integrated decoy’ or as a domain with an altogether different function.

In plant genomes, NLR loci are often found as complex multi-loci regions with allelic series ranging from moderate to extreme sequence divergence [25,26,27,28,29]. In addition, all characterized NLR-IDs to date have been shown to work as pairs with a canonical NLR that is required for activation of defense response upon pathogen recognition. These include RRS1(WRKY)/RPS4 in *Arabidopsis thaliana*, as well as *RGA5*(HMA)/*RGA4*, *Pik1*(HMA)/*Pik2,* and *Pii-2*(NOI/RIN4)/*Pii-1* in rice [5,6,7,8,10] (the ID is indicated for the corresponding NLR-ID). The partner NLR is invariably located within 5 kb of the NLR-ID and both genes are in a head-to-head orientation (i.e., they share a common promoter region) [7,8,10,30]. These features can be investigated by exploring the genomic regions surrounding the NLR-ID, however, this task is made difficult by the lack of contiguous sequence assemblies for accessions with the functional NRL-IDs. Not all NLR-IDs identified in genome-wide studies are located in head-to-head orientation with another locus [11], suggesting that either this feature is characteristic of the functional NLR-IDs or certain NLR-IDs may function according to a model that is different to the ‘Integrated Decoy’ model. For example, several cases of NLR detecting the pathogen (sensors) require a second locus for initiating immune response signaling (helper) that is not necessarily located in close proximity. These include the NLR-REQUIRED FOR CELL DEATH (NRC) network in Solanaceae [31] and networks mediated by ACTIVATED DISEASE RESISTANCE 1 (ADR1) [32,33] and N REQUIREMENT GENE 1 (NRG1) [34,35].

We previously defined the synteny across wheat and related grasses in the *Yr7/Yr5* region [18]. We showed an expansion in the number of BED-NLR loci in the *Triticum aestivum* reference genome (landrace ‘Chinese Spring’; RefSeqv1.0) and wild emmer wheat *T. turgidum* ‘Zavitan’ when compared to related grass species [18]. We identified highly similar sequences to *Yr5a* (originally from spelt wheat) in UK elite wheat varieties, in addition to the functional *Yr7* sequence present in Cadenza. However, the lack of contiguity in these UK wheat genome assemblies made it difficult to determine whether the *Yr5a* related sequences were within the *Yr7/Yr5* region and if the functional *Yr5a* and *Yr7* genes had additional NLR loci in their vicinity. 

Recently, chromosome-level assemblies from nine bread wheat cultivars, and a spelt cultivar, were produced as part of the 10+ Wheat Genome Project [36]. Here we used these chromosome-level assemblies to determine the genomic architecture of the *Yr7/Yr5* region across modern wheat varieties and determine the presence of putative partner NLRs in the vicinity of the functional *Yr5a* and *Yr7* genes. We observed that BED domains from BED-NLRs show sequence similarities with BED domains from single-BED proteins in 20 plant species containing BED-NLRs. Using transient assays in *N. benthamiana*, we tested whether Yr7 carries a functional NLS downstream of the BED domain and the ability of the Yr7 protein to induce cell-death in this heterologous system. Together, these results improve our understanding of the potential role of the BED domain in BED-NLRs and will help prioritize future functional studies.

## 2. Materials and Methods 

### 2.1. Yr7 and Yr5 Alleles Identification in the Multiple Wheat Cultivar Genomes

We used the *Yr7* and *Yr5a* (syn. *Yr5*) sequences from Marchal et al (2018) [18] to carry out BLASTn analyses [37] in the *T. aestivum* genomes listed in Table 1. The *Yr7* sequence is derived from hexaploid wheat *T. aestivum* cultivar Cadenza (Cadenza-*Yr7*, Genbank MN273771.1), whereas the *Yr5a* sequence is from hexaploid spelt wheat *T. aestivum* ssp. *spelta* cultivar Album (Album-*Yr5*, Genbank MN273772.1). We retrieved hits that covered at least 90% of the *Yr7* or *Yr5a* sequence and considered hits sharing more than 99% identity with either *Yr7* or *Yr5a* as potential alleles, provided they are located in the *Yr* region. We describe the best hits to *Yr7* and *Yr5a* in Supplementary Figure S1.

### 2.2. Definition of Syntenic Regions across Wheat Cultivar Genomes

**Figure 1:**
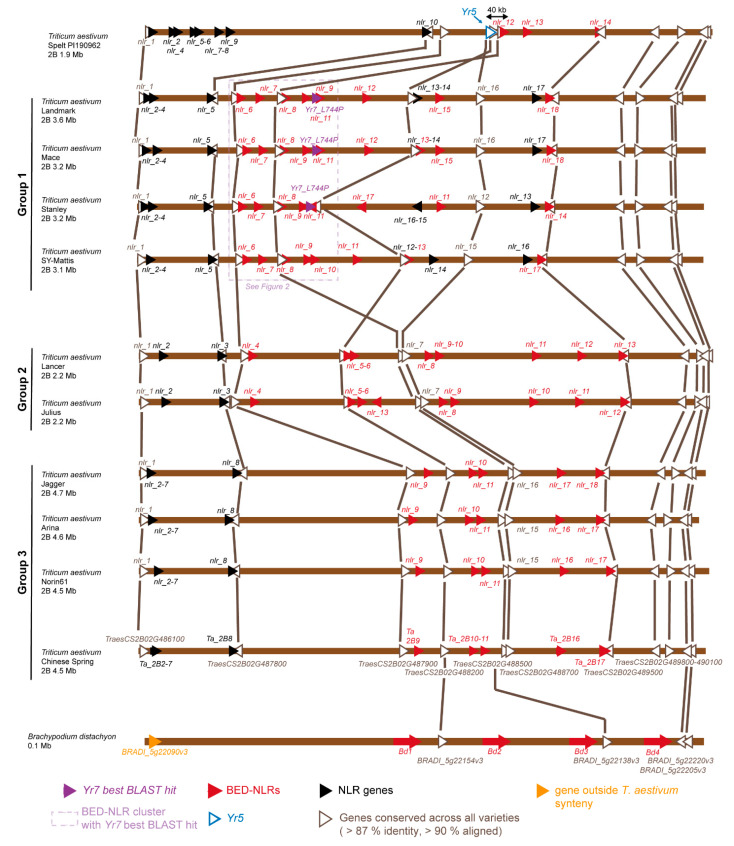
Schematic representing the *Yr* region in ten wheat varieties, a spelt variety and *B. distachyon*. The syntenic region was defined based on the eleven gene models with hits across all varieties (gene models shown as white triangles). Dark brown represents the region of chromosome 2B in synteny with the *Yr* region on RefSeqv1.0. Black triangles show potential canonical NLR loci annotated with NLR-Annotator and red triangles potential BED-NLR loci (Table S3). Purple triangles depict *Yr7* best BLAST hit in Group 1 varieties (*Yr7_L477P*) and the white triangle with a blue outline shows *Yr5a* in spelt. The BED-NLR-rich region in the vicinity of the *Yr7* is highlighted with a dashed light purple line (see Figure 2 for details). The orientation of the triangles reflects the orientation of the gene model. The orange triangle in *B. distachyon* represents a gene that was outside of the synteny with wheat. NLRs sharing 100% identity across 95% of their sequences are reported in Table S4.

We extracted all RefSeqv1.1 gene models [38] located with the *Yr* syntenic region between TraesCS2B02G486000 and TraesCS2B02G490200 (defined in Marchal et al. (2018) [18]) and performed BLASTn analyses against the wheat assemblies described in Table 1. We filtered out hits covering less than 90% of the query sequence and sorted the BLAST results by: (1) Bitscore, (2) e-value, and (3) percentage identity (Supplementary Table S1). We retained only the top hit for each RefSeqv1.1 gene model and used the percentage identity value to generate the heatmap presented in Supplementary Figure S2. We used the heatmap.2 function of the ggplot2 R package v3.2.0. to produce the heatmap [39]. We retrieved the 11 gene models that had hits in all genome assemblies and used these to determine the location of the *Yr* region in the wheat assemblies studied here (Figure 1). Four RefSeqv1.1 gene models (TraesCS2B02G488200, TraesCS2B02G488500, TraesCS2B02G490000 and TraesCS2B02G490100) had hits with more than 80% sequence identity with *B. distachyon* genes and were located in the previously defined syntenic *Yr* region (Figure 1) [18].

#### 2.2.1. Definition of the Yr Region Gene Content in the Multiple Wheat Cultivar Genomes

There is currently no de novo gene annotation available for the ten newly sequenced and assembled wheat genomes listed in Table 1. We therefore used projections of the RefSeqv1.1 (Chinese Spring) gene models onto these ten assemblies. These projections combine both sequence similarity and synteny information to assign a RefSeqv1.1 gene model to a specific position on the ten genomes. On average, 99% of all Chinese Spring high-confidence genes were successfully transferred to the ten wheat genomes.

#### 2.2.2. Definition of the NLR Content of the Syntenic Regions in the Wheat Genomes

**Figure 2:**
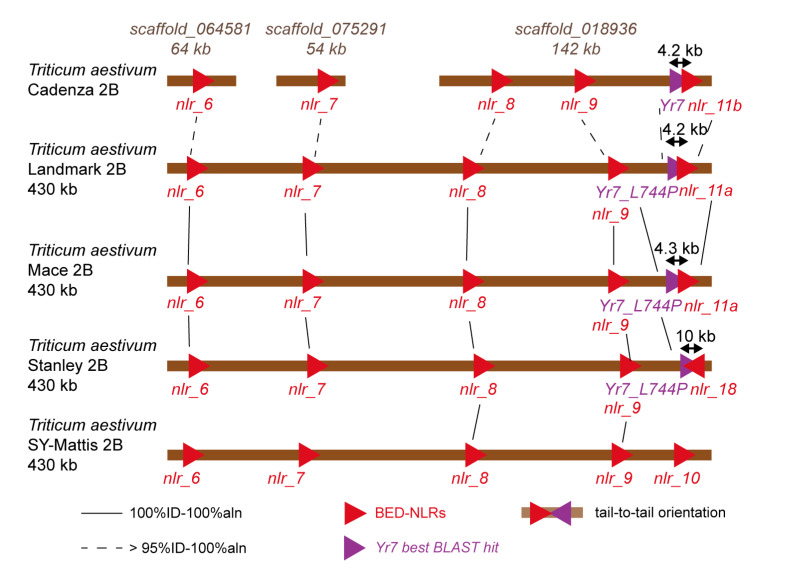
Close-up on the BED-NLR-rich region in Landmark, Mace, Stanley and SY-Mattis where *Yr7-L744P* is located. Colours and shapes shown in this figure correspond to those used in Figure 2. We added the scaffolds of the Cadenza assembly that contained hits to the Landmark BED-NLRs, including the scaffold which contains *Yr7*. The orientation of the triangles reflects the orientation of the loci. We identified a BED-NLR locus in tail-to-tail orientation located 10 kb from Stanley-*Yr7-L744P*. In Cadenza, Landmark and Mace, we identified *nlr_11a/b* that was located ~4.5 kb and in tail to head orientation with *Yr7*/*Yr7-L744P*.

We used NLR-Annotator [40] to identify potential NLR loci in each of the syntenic regions across the ten genome assemblies. NLR-Annotator identifies NLR-related motifs in genomic sequences and predicts putative loci based on the distance and presence/absence of these motifs. It does not predict Open Reading Frames (ORFs), rather regions that may harbor NLR loci. All NLR sequences predicted by NLR-Annotator and located in the *Yr* region are available in File S1. We performed a multiple sequence alignment of all NLR sequences with Clustal Omega (https://www.ebi.ac.uk/Tools/msa/clustalo/ [41]) and generated a percentage identity matrix (Supplementary Table S2) and associated heatmap (Supplementary Figure S3) to represent sequence similarity across all NLRs found in the *Yr* region. We looked for putative NLR loci overlapping with a projected gene model and kept the gene structure derived from the gene model when possible [Table S3]. Some translated proteins derived from these projections contained premature termination codons, suggesting that there might be differences between the Chinese Spring gene structure and their best hits in the wheat genome assemblies from Table 1. We identified the potential NLR alleles across the multiple wheat assemblies when a hit had 100% identity over at least 100% of the sequence of the query [Table S4]. A close-up of the BED-NLR-enriched region containing the *Yr7* best hits in Landmark, Mace and Stanley is presented in Figure 2.

When no projected gene model was identified for a given cultivar, we carried out a 6-frame translation (https://www.ebi.ac.uk/Tools/st/emboss_transeq/) of the extended (±1000 bp) NLR-annotator loci. We subsequently used hmmscan from HMMER v3.1 [42] to compare these sequences with the Pfam database (ftp://ftp.ebi.ac.uk/pub/databases/Pfam) and identify additional domains, such as BED domains, in the gene models and the translated sequences (Supplementary Table S3). We applied a 0.01 i-evalue cut-off to filter out irrelevant identified domains. This allowed us to determine NLR proteins which were likely to be BED-NLRs and canonical NLRs.

#### 2.2.3. Annotation of nlr_11 in Cadenza

We performed a BLAST analysis with *nlr_11* from Landmark and Mace (*nlr_11a*) against the Cadenza whole-genome assembly (https://opendata.earlham.ac.uk/opendata/data/Triticum_aestivum/EI/v1.1/) to determine whether Cadenza also carries *nlr_11* in the vicinity of the *Yr7* locus. We identified a hit sharing 98% sequence identity with *nlr_11* from Landmark/Mace, with all polymorphisms restricted to the 3’ end of the sequence (Supplementary File S2). In addition, the *nlr_11* sequence in Cadenza included a large region containing ‘Ns’ within the putative coding sequence, which we corrected using PCR amplification of Cadenza and subsequent Sanger sequencing (primers and curated sequence in Supplementary File S2). 

To annotate the gene structure of *nlr_11* in Cadenza (*nlr_11b* hereafter), we used HISAT2 [43] (v2.1) to map RNA-Seq reads from non-infected leaf tissue derived from Cadenza seedlings (3-leaf stage, SRA accession PRJNA603450) to the Cadenza genome assembly containing the curated *nlr_11b* sequence (Supplementary File S2). We used the following parameters: --no-mixed --no-discordant to map reads in pairs only. We used the --novel-splicesite-outfile to predict splicing sites that we manually scrutinized with the genome visualization tool IGV (v2.3.79) [44]. Predicted coding sequences (CDS) were translated using the ExPASy online tool (https://web.expasy.org/translate/). We show a schematic of the *nlr_11* gene structure in Supplementary Figure S4.

### 2.3. Identification of BED-NLRs and BED-proteins in Plant Genomes

We downloaded 90 plant proteomes from Phytozome v12.1 (https://phytozome.jgi.doe.gov/pz/portal.html) and EnsemblPlants (https://plants.ensembl.org/index.html) (Table S5) and identified complete Benchmarking Universal Single-Copy Orthologs (BUSCO) with the BUSCO program (v3) [45]. Given that we investigated proteomes from all plant kingdom, we performed two BUSCO analyses: one with the Viridiplanteae set [46], which comprises 430 orthologs, and one with the Embryophytes set [46], which comprises 1440 orthologs. We filtered-out any proteome displaying less than 90% of complete orthologs from the Viridiplanteae set and any Embryophyte proteome displaying less than 90% of complete orthologs from the Embryophyte set. Our final set contained 68 proteomes (69 when including RefSeqv1.0, Supplementary Table S5).

For these 69 proteomes, we identified proteins carrying a BED domain with HMMER (v3.1) and the Pfam database as described previously. We retained a total of 20 proteomes containing both BED-NLRs and other BED-containing proteins for the Neighbor-net analyses (Supplementary Table S5). We separated the set between NLR and non-NLRs based on the presence of the NB-ARC domain. BED domains were extracted from the corresponding protein sequences based on the HMMER output (Supplementary Table S6).

### 2.4. Neighbor-net Analyses

**Figure 3:**
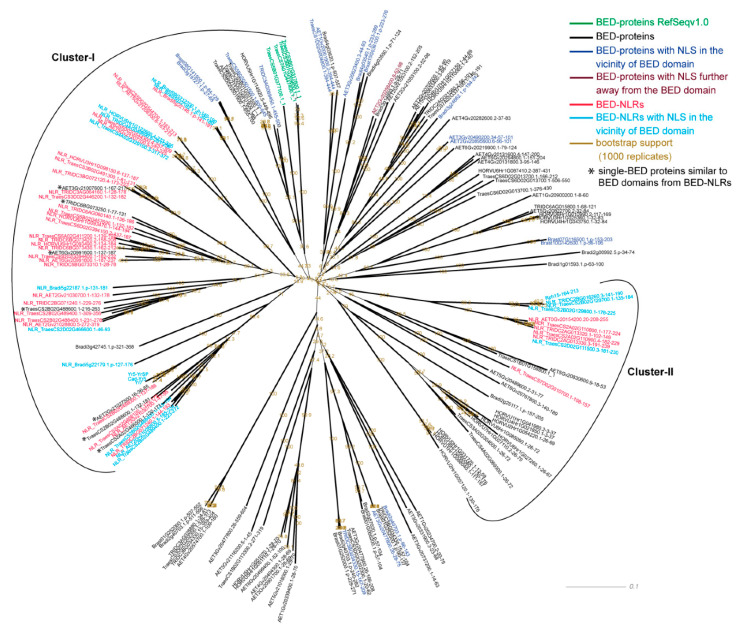
Neighbour-network of BED domains from non-NLR and NLR proteins in the Pooideae. The network was generated with SplitsTree v.4.16 [48] from a multiple sequence alignment produced with MAFFT v7.305 and the L-INS-I method [53]. Bootstrap values are reported in gold, NLR proteins in red and non-NLR proteins in black. Protein carrying a nuclear localization signal (NLS) in the vicinity of the BED domain (within 40 amino-acid up or downstream) are depicted in light blue (NLRs) and dark blue (non-NLRs), whereas proteins carrying an NLS further away from the BED domain are shown in brown (non-NLRs). The full list of NLS-containing proteins is in Table S15. Protein that were annotated as BED-DUF4413-(hAT) in RefSeqv1.0 gene models but are annotated as single-BED in RefSeqv1.1 gene models are shown in green. Asterisks indicate BED-only proteins that share sequence similarities with BED domains from BED-NLRs (Tables S6 and S8). Clusters were defined based on the presence of BED-NLRs and the closest clusters harboring non-NLR proteins (see Results).

We previously reported that given that BED domains (PF02892) are short (45 amino acids on average) and highly variable, they generate conflicting phylogenetic signals [18]. As a result, there is no single tree topology showing high bootstrap support in the phylogeny analyses we conducted on BED domains derived from different BED-containing proteins. To address this, we used the Neighbor-net method [47] implemented in SplitsTree4 (v4.16) [48] to analyze the degree of similarity between BED domains from NLR and non-NLR proteins in wheat and related Pooideae, without inferring evolutionary relatedness (Figure 3). 

We first retrieved all BED-containing proteins (BED-NLR and non-NLR proteins) from RefSeq v1.0 [38], *Triticum dicoccoides* [49], *Aegilops tauschii* [50], *Hordeum vulgare* [51], *B. distachyon* [52] and *B. stacei* (DOE-JGI, http://phytozome.jgi.doe.gov/). We performed a multiple sequence alignment with MAFFT v7.305 and the L-INS-I method [53] and generated a neighbor-network in SplitsTree4 based on the uncorrected P distance matrix between sequences (Figure 3). Although SplitsTree produces a distance matrix when we queried unaligned sequences, it is important to note that SplitsTree does not produce a real alignment by default but adds gap at the end of the sequences to make them all have the same length (Noalign method, http://nebc.nerc.ac.uk/bioinformatics/documentation/splitstree/manual.pdf). We thus first used MAFFT to generate a multiple sequence alignment with the BED domains and used this as an input file in SplitsTree. We included the BED domain sequences of Yr7, Yr5a/Yr5b, Cadenza-Yr5, and Rph15 (GenBank MT385775) that are not present in the reference assemblies analyzed here. We carried out identical analyses in the 14 additional proteomes containing BED-NLRs and BED proteins, including the BED domain sequence of Xa1, which is identical to that of the *Xo1* [16] (Supplementary Figures S5–S8). All the amino-acid sequences of the retrieved BED domains can be found in Supplementary File S3. Given the high variability of the BED domain, we grouped together species that were close phylogenetically to increase the power of the analysis. This allowed us to identify BED domains from non-NLR proteins that were (i) interspaced with BED domains from BED-NLRs, thus sharing relatively high sequence similarity and (ii) belonged to the closest group of BED domains from BED-NLRs, thus being slightly more divergent (Supplementary Tables S7–S10). For example, in the Pooideae, cluster I was well defined with the majority of proteins being BED-NLRs (Figure 3). However, in the case of cluster II, fewer BED domains from BED-NLRs were present and we included sequences from the closest group to these BED-NLRs (See result section and Figure 3).

We defined the protein architecture of the flanking gene models of non-NLR proteins that were interspaced with BED-NLRs in the neighbor-network to determine whether these could be truncated BED-NLRs (Supplementary Tables S7 and S8). Most of the non-NLR proteins located in the vicinity of NLR-related genes were gene encoding BED domain(s) only (Supplementary Tables S7 and S8). We refined this analysis and extracted 21 kb up- and downstream all genes encoding BED domain(s) only and determined the presence of NLR-related motifs in these regions with NLR-Annotator [40,54] (Supplementary Tables S9 and S10). Additionally, we performed BLAST analyses to determine whether the BED domains from these proteins encoding BED domain(s) only shared sequence similarities with BED domains from BED-NLRs (Supplementary Tables S9 and S10). This allowed us to report gene encoding BED domain(s) only that could be truncated BED-NLRs and thus should be removed from the enrichment analysis described below. We labelled these potential truncated BED-NLRs with an asterisk in Figure 3 and Supplementary Figures S5–S8.

To determine whether specific BED-proteins were more likely to cluster with BED-NLRs based on BED domain similarity, we retrieved all additional domain identified in the HMMER analysis (Supplementary Tables S11–S13). We carried out an exact Fisher’s test to determine whether the proportion of a given domain in BED-protein clustering with BED-NLRs was significantly higher than the proportion of this domain in BED-proteins in general (*p*-value < 0.05, Supplementary Table S14).

### 2.5. Identification of Nuclear Localisation Signal (NLS) in BED-containing Proteins and Yr7 Cellular Localisation Experiments

We used NLSdb267 (https://rostlab.org/services/nlsdb/) to predict NLS in BED-containing proteins and then determined their distance to the BED domain (Supplementary Table S15, Supplementary Figure S9). The alignments presented in Supplementary Figure S9 were performed with MAFFT [53] v7.305 using default parameters. We visualized the alignments with Jalview [55] v2.10.1.

#### 2.5.1. Generation of the Constructs for Yr7 Cellular Localisation Experiments in N. Benthamiana

**Figure 4:**
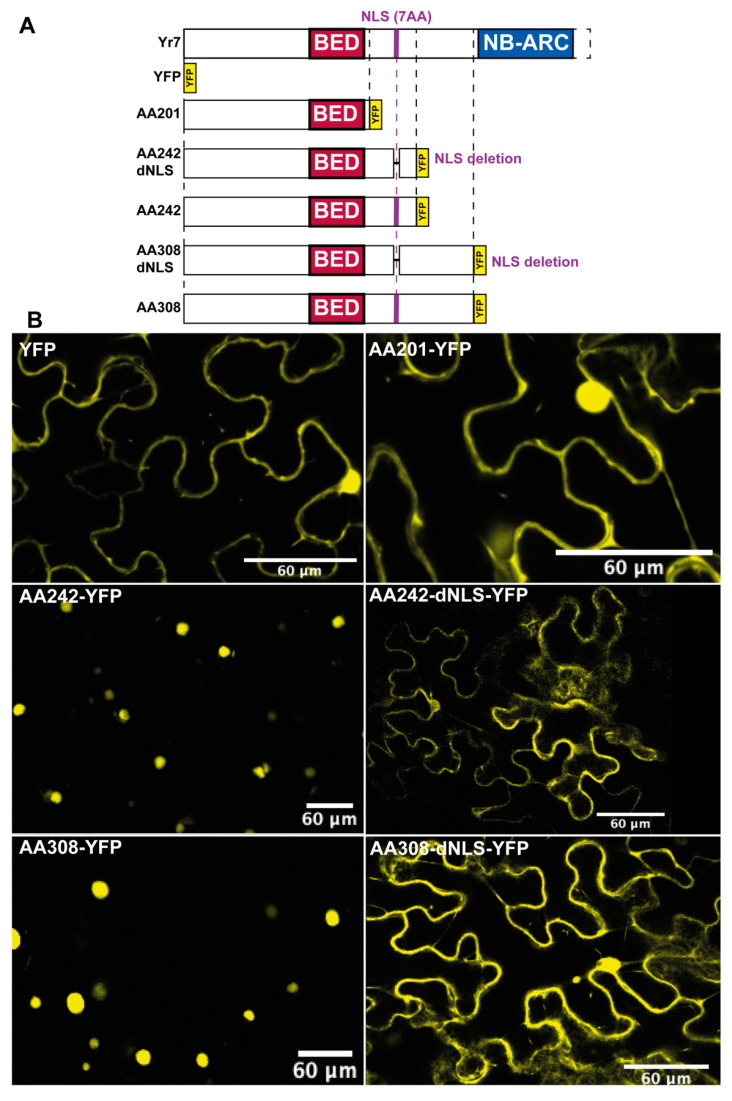
Cellular localization in *N. benthamiana* of truncated variants of the Yr7 protein and their corresponding deletion mutants in the predicted NLS. (**A**) Schematic describing the different truncations analysed and their corresponding deletion mutants in the predicted NLS (purple). Yr7-AA201 does not contain the predicted NLS. Both Yr7-AA242 and AA308 contain the predicted NLS whereas Yr7-AA242-dNLS and Yr7-AA308-dNLS are mutants lacking the predicted NLS. (**B**) Fluorescent microscopy pictures from leaves expressing the corresponding Yr7 variant. Samples were taken at 1.5 dpi and five leaf fragments from two leaves per plant (two plants per construct) were assessed under confocal microscope (see Methods section). Single YFP was used as a negative control.

We used GoldenGate cloning [56] to generate the constructs used for the Yr7 cellular localization experiments described on Figure 4. We synthesized *Yr7* cDNA sequence (4761 bp) from the Cadenza cultivar and removed *Bpi*I and *Bas*I restriction sites using the genetic code to avoid generating missense mutations. We cloned this synthesized fragment into the level 0 acceptor pUAP1 (Addgene plasmid #63674) [57] to generate a level 0 module for Golden Gate cloning lacking its STOP codon (Addgene plasmid #141093). We synthesized an additional *Yr7* cDNA codon-optimized for *N. benthamiana* protein expression (Genewiz). We cloned this synthesized fragment into the level 0 acceptor pUAP1 (Addgene plasmid #63674) [57] to generate a level 0 module for Golden Gate cloning lacking its STOP codon (Addgene plasmid #141092). Details of the level 1 constructs generated from the codon-optimized and non-codon-optimized versions are available in Supplementary Table S16 and Supplementary File S4.

We generated a total of six constructs corresponding to the Yr7 recombinant proteins harboring a yellow fluorescent protein (YFP) tag in the 3’ terminus and a YFP transcription unit (File S4). The first three corresponded to different truncations of the Yr7 protein. These all initiated from the start Methionine and included sequence up to amino acid position 201 (AA201), position 242 (AA242), or position 308 (AA308). In addition, we generated two deletion mutants for the AA242 and AA308 constructs in which the NLS (SNGKRKR: amino acid positions 219 to 225) were excluded from the sequence (primers listed in Supplementary Table S16 and plasmid maps in Supplementary File S4). Each truncated version of Yr7 was cloned in level 0 acceptor pUAP1 (Addgene #63674). Level 1 transcription units were generated with the GoldenGate modules 35S + Ω promoter (pICH51266, AddGene #50267), the Yr7 truncations in pUAP1 described above, YFP tag (pICSL50005) and 35S CaMV terminator (pICH41414, AddGene #50337) in the level 1 acceptor pICH47742 (AddGene #48001). Additionally, a YFP transcription unit was assembled with the same regulatory elements and the YFP CDS pAGM3212. GoldenGate modules were obtained from the TSLsynbio database (http://synbio.tsl.ac.uk/) [56,57,58].

#### 2.5.2. Generation of the Constructs for Yr7 Hyper-sensitive Response Assays in N. Benthamiana

We tested whether *N. benthamiana* could be used as a heterologous system to study Yr7 mediated cell-death signaling (Supplementary Figure S10). We infiltrated *N. benthamiana* leaves with *A. tumefaciens* clones transformed with the constructs described above: full-length Yr7 protein, the different Yr7 truncations and additional truncations in the N-terminus of the Yr7 protein (Supplementary Table S16, Supplementary File S4). Given that amino-acid substitutions in the ‘MHD’ motif of several NLRs that can lead to constitutive cell-death signaling in *N. benthamiana* [59,60,61], we generated ‘MHD’ variants of the Yr7 protein with the aim to generate an ‘auto active’ Yr7 variant (Supplementary Table S16, Supplementary File S4). 

We developed the constructs using Golden Gate cloning as described above. We generated Yr7-exon1 (AA1-100) and Yr7-BED (AA136-186) from the non-codon optimized version of *Yr7* cDNA (Addgene plasmid #141093) using the primers listed in Table S16 and cloned these sequences in level 0 acceptor pUAP1 (Addgene #63674). Level 1 transcription units were generated with the same promoter, C-terminal tag, and terminator modules as described above (pICH51266-AddGene #50267, pICSL50005 and pICH41414-AddGene #50337). We developed a D646V mutant in the ‘MHD’ motif of the Yr7 sequence using the corresponding primers described in Table S16 and cloned this variant in level 0 acceptor pUAP1 (Addgene #63674). We generated level 1 transcription unit the same way as for Yr7-exon1 and Yr7-BED, however, we used a 6xHA tag (pICLS50009A) at the C-terminus of the protein to monitor protein expression in planta. We used Mla10-HA recombinant protein as a positive control [62] and Pikp2-HA [63] as a negative control for HR signaling in *N. benthamiana*. Mla10-HA was provided by Dr. Hiroaki Adachi (Sainsbury Laboratory) and Pikp2-HA by Dr. Thorsten Langner (Sainsbury Laboratory). 

#### 2.5.3. Transient Assays in N. Benthamiana

We transferred the Yr7 constructs described above, in addition to YFP, Mla10 and Pikp-2 control constructs into *A. tumefaciens* GV3101 via electroporation. An overnight 10 mL liquid culture (LB medium + selection antibiotic) of *A. tumefaciens* carrying the plasmid was prepared for infiltration in 4–6 weeks old *N. benthamiana* plants. We prepared the *A. tumefaciens* suspension in infiltration buffer (10 mM MES, 10 mM MgCl2, and 150 μM acetosyringone, pH 5.6) and we adjusted the OD600 to 0.5. Additionally, we co-infiltrated the full-length Yr7 transformant with an *A. tumefaciens* expressing p19, the suppressor of posttranscriptional gene silencing of tomato bushy stunt virus that is known to enhance in planta protein expression (pICSL11155, [64]). We adjusted the OD600 to 0.1 for the p19 strain prior infiltration.

In the cellular localization experiments, we infiltrated two whole leaves per plant in a total of two plants (i.e., four infiltrations) and carried out the experiment three independent times to validate cellular localization. Infiltrated *N. benthamiana* leaves were harvested 1.5 days post infiltration and kept on imbibed filter paper until observation under confocal microscope (Leica SP5). Five fragments from each leaf were cut with a scalpel and mounted in water on microscope slides. Argon ion excitation laser (514 nm) was used to observe YFP-related fluorescence in the samples. Observations were done with ×10 and ×20 objectives.

For the hypersensitive response (HR) assays, we infiltrated each transformed *A. tumefaciens* in defined zones of each leaf as showed in Supplementary Figure S10. For each leaf pattern, we infiltrated two leaves per plants in a total of two plants (i.e., four infiltrations) and carried out the experiment three independent times to validate the HR phenotype. We recorded the HR phenotype between five- and seven-days post-infiltration. For the protein expression experiments (immunoblots), we infiltrated two whole leaves per plant with a given *A. tumefaciens* transformant and carried out protein extractions at 1, 2, 3- or 4-days post-infiltration depending on the tested construct (Supplementary Figure S10). We pooled the two infiltrated leaves for protein extraction. Samples were prepared in a 1:2 weight:volume of protein extraction buffer (10% glycerol, 25mM Tris pH 7.5, 1mM EDTA, 150mM NaCl, 10 mM DTT, 2% w/v PVPP, protease inhibitor cocktail (SIGMA #P9599), 0.2% IGEPAL (SIGMA #I3021)). We centrifuged the samples 3000× *g* for 10 min at 4 °C and filtered the supernatant in 2.5 mL syringes with 250 µm filters. We centrifuged the filtrate 13,000× *g* rpm for 10 mins and used 20 µL of the supernatant for SDS-PAGE. Immunoblotting was performed with HA-probe (F-7) HRP (Santa Cruz Biotech) or anti-GFP antibody (ab290, abcam) in a 1:5000 dilution.

## 3. Results

### 3.1. Identification of NLRs Across the Yr7/Yr5 Locus in the Multiple Wheat Cultivar Genomes

Well-characterized NLR-IDs work in pairs whose partners are in close proximity (<5 kb) and harbor a head to head orientation in the genome [7,8,10,30]. We investigated long-range assemblies of 10 wheat cultivars/accessions to determine whether they carry loci that are similar and syntenic to *Yr7* and *Yr5a*, and if paired NLR-IDs exist in their vicinity. We focused on BLAST hits that covered at least 90% of the functional Album-*Yr5a* or Cadenza-*Yr7* sequences (referred to as *Yr5a* and *Yr7* below, respectively) (Table 2). 

**Table 2:** In silico allele mining for Cadenza-*Yr7* and Album-*Yr5a* in the ten chromosome-quality wheat assemblies.

Genome	Identity to Album-*Yr5a* on 2B (%)	Higher Identity to Album-*Yr5a* on Other Chromosomes (%)	Identity to Cadenza-*Yr7* on 2B (%)	Higher Identity to Cadenza-*Yr7* on Other Chromosomes (%)
Arina	-	**99.31 (2D) ^1^**	86.5	-
SY-Mattis	-	**99.31 (2D) ^1^**	-	-
Julius	95.1	**99.75 (2D) ^2^**	-	-
Jagger	-	**99.75 (2D) ^2^**	86.5	-
Lancer	95.1	-	-	-
Stanley	-	-	**99.98**	-
Mace	-	-	**99.98**	-
Landmark	-	-	**99.98**	-
Chinese Spring	-	-	86.5	-
Norin61	-	-	86.5	-
Spelt PI190962	**100**	-	-	-

^1^ Chromosome 2D sequence identical to Cadenza-*Yr5.* ^2^ Chromosome 2D sequence identical to Claire-*Yr5.* Percentage identities are recorded from the start to the stop codons of Cadenza-Yr7 and Album-Yr5a, including intron sequences. Only hits covering more than 90% of the length of Yr7/Yr5a are reported and we highlighted in bold and underlined hits sharing more than 99% identity with Cadenza-Yr7 or Album-Yr5a.

We identified an identical hit to Album-*Yr5a* in the spelt accession PI190962 on chromosome 2B (Table 2), suggesting that PI190962 carries the functional *Yr5a* allele. There was no other sequence that was both highly similar to *Yr5a* (sharing at least 99% identity across 90% of the sequence of *Yr5a*) and located on chromosome 2B (Table 2). Julius and Lancer both carry a sequence that shares 95.1% identity with *Yr5a* over 92% of its sequence. The previously identified *Yr5a* hit in Chinese Spring was below the cutoff (only 86.5% identity across 81% of *Yr5a* sequence). Additionally, the two previously described *Yr5a* hits to Cadenza and Claire [18] were also identified, however, they were assigned to chromosome 2D in Arina/SY-Mattis and Julius/Jagger, respectively. Given the more contiguous nature of the new assemblies it is likely that these loci are located on chromosome 2D.

In the case of *Yr7*, no variety carries the functional Cadenza-*Yr7* allele. We did, however, identify a very close hit in Landmark, Stanley and Mace, sharing 99.98% sequence identity (Table 2). A single nucleotide polymorphism common in these three varieties (T2449C), leads to a non-synonymous amino-acid substitution in an LRR repeat (L744P) (Supplementary Figure S1). The functional consequence of this mutation is unknown. Additional hits in Arina, Jagger, Norin61, and Chinese Spring share 86.5% sequence identity with Cadenza-*Yr7,* over 94% of its sequence. 

We next asked whether the closest hits to *Yr7* and *Yr5a* were in syntenic position with the previously defined *Yr7/Yr5a* region (between TraesCS2B02G486000 to TraesCS2B02G490200 [18]). We retrieved all annotated genes on RefSeqv1.1 (Chinese Spring) across this interval and performed BLAST analyses to assess the diversity and locate the corresponding sequences across the additional wheat varieties (Figure S2 and Figure 1, Table S1). Using the criteria that only hits covering > 90% of the gene model length were retained, we identified high diversity in the region across varieties. We defined three groups of varieties, with Chinese Spring, Arina, Jagger and Norin forming one group, Landmark, Stanley, Mace and SY-Mattis forming another group, and Julius and Lancer forming the last group (Figure 1 and Figure 2). The spelt accession was distinct (Figure 1, Table S1). Of the 42 queried gene models, only eleven had a BLAST hit across all studied genomes (covering at least 90% of RefSeqv1.1 gene models) (Supplementary Table S1, Figure 1 and Supplementary Figure S2). These eleven RefSeqv1.1 gene models were near-identical to Chinese Spring in Norin, Arina and Jagger (Table S1, Figure S2) and most dissimilar in Spelt PI190962 (e.g., TraesCS2B02G489900 shared only 87.4% sequence similarity with the corresponding sequence in spelt). Interestingly, two of these conserved hits corresponded to NLR loci. TraesCS2B02G486100 encodes NB-ARC and LRR domains (1280 amino acids) and TraesCS2B02G488700 encodes LRRs only (480 amino acids). Using these eleven common loci, we defined the *Yr7/Yr5a* region across each variety. These intervals encompassed the sequences with the highest identities to *Yr7* and *Yr5a* described in Table 2 (Supplementary Figure S2).

We next investigated potential paired NLR-IDs in close proximity to the *Yr7* and *Yr5a* strongest hits across the *Yr7/Yr5a* regions of each variety. For Album-*Yr5a* we had previously identified an identical allele in spelt PI190962. Here, the closest NLR is located 40 kb distal to *Yr5a* (Figure 1); this is farther to what has been described for NLR pairs (<5 kb, [7,8,10,30]). For *Yr7*, we focused on Stanley, Mace and Landmark which have the *Yr7*-L744P allele with the single amino-acid substitution in the LRR repeat. These loci are part of a wider conserved BED-NLR cluster across these varieties, which also include SY Mattis (Figure 2). For Stanley, we defined a predicted NLR (*nlr_18*) located 10 kb distal to *Yr7*-L744P and in tail-to-tail orientation. This is thus different from what is expected from characterized paired NLRs (Figure 2). The *nlr_18* locus overlaps with the projected Stanley gene model TraesSTA2B01G533400 (Supplementary Table S3). However, when compared to the original RefSeqv1.1 gene model TraesCS2B02G488400, the *nlr_18* ORF is disrupted (Supplementary File S2). It is important to bear in mind that the projected RefSeqv1.1 gene models may not reflect the actual structure of the gene in the targeted varieties. Additional data, for example RNA-seq data from Stanley, would be required to confirm the gene structure and determine whether this locus encodes a full-length protein.

For Landmark and Mace, we identified an NLR locus (*nlr_11*) located < 4.5 kb from the *Yr7*-L744P allele (Figure 2), although again not in head to head orientation. A similar organization is observed in the Cadenza assembly with the functional *Yr7* allele 4.2 kb proximal to *nlr_11* (Figure 2, Supplementary File S2). However, given that there was a large region containing ‘Ns’ in the Cadenza hit, we corrected the sequence using Sanger Sequencing (Supplementary File S2). The corrected *nlr_11* sequence in Cadenza shares 97.7% identity with *nlr_11* in Landmark/Mace (Supplementary File S2). We thus named these loci *nlr_11a* and *nlr_11b* in Landmark/Mace and Cadenza, respectively. As no projected gene models overlapped with *nlr_11a* in Landmark and Mace, we used RNA-seq reads from Cadenza to annotate *nlr_11b* in this variety (Supplementary Figure S4, Supplementary File S2). We identified three putative exon sequences, with defined canonical splice donor and acceptor sites, and used this to predict a protein sequence. This sequence included a BED and NB-ARC domains. However, both exon 2 and 3 contain multiple in frame premature termination codons (Supplementary Figure S4). We used the gene structure of *nlr_11b* in Cadenza to infer the potential gene structure of *nlr_11a* in Landmark/Mace (Supplementary File S2). Across the putative coding region covered by RNA-Seq reads, both *nlr_11a* and *b* were identical. Hence, the gene structure is most likely shared between these loci. This suggests that the predicted *nlr_11* sequence in Cadenza and Landmark/Mace is a pseudogene that generates a truncated protein with a partial BED domain. In summary, these results reveal a highly dynamic BED-NLR cluster in wheat, but no presence of a functionally relevant paired NLR gene architecture for either *Yr5a* nor *Yr7* or its closely related *Yr7*-L744P allele (Figure 2).

### 3.2. The Yr Region is Conserved across the Multiple Wheat Cultivar Genomes

We previously highlighted an expansion of the number of BED-NLRs within the wider *Yr7/Yr5* region in hexaploid wheat Chinese Spring when compared to D-genome donor *Aegilops tauschii*, barley (*Hordeum vulgare)*, *Brachypodium distachyon* and rice (*Oryza sativa*) [18]. We therefore sought to determine whether this observation was consistent across the additional accessions of the wheat genome. The *Yr* region varied in physical size from 1.9 Mb in Spelt PI190962 to 4.7 Mb in Jagger (Figure 1, Table 3). We defined three major groups of hexaploid wheat varieties based on the degree of conservation in both the architecture and the gene sequences of the *Yr* region (Figure 1, Supplementary Table S4). Group 3, which includes RefSeqv1.1 (Chinese Spring [38]), showed an architecture very close to that observed in Group 2 (Julius/Lancer) based on the position of the eleven conserved gene models described in the previous section (Figure 1). In Group 1 varieties, which include Landmark, Mace and Stanley (*Yr7*-L744P), and SY-Mattis, there was a rearrangement involving TraesCS2B02G488500 and TraesCS2B02G488200 compared to Group 2 and 3 varieties. Unique among Group 1 varieties, Stanley has an inversion involving TraesCS2B02G488200 and additional NLR loci, including *nlr_11b* but not *Yr7-L744P*. In Spelt PI190962, we identified rearrangements in this same region (TraesCS2B02G488200, TraesCS2B02G488500, and TraesCS2B02G488700) and a reduction in size of the region between these loci when compared to the other varieties. Using the previously defined syntenic region in *B. distachyon* and based on TraesCS2B02G488200 and TraesCS2B02G488500, we defined Groups 2 and 3 (including Chinese Spring) gene order to be ancestral (Figure 1). The *Yr* region is thus overall syntenic, but variable in terms of organization and architecture in the investigated wheat varieties.

We next investigated the gene content of the *Yr* region across the multiple wheat cultivar genomes. Based on the projection of Chinese Spring gene model onto the wheat genome assemblies, we identified between 32 to 54 projected gene models within the region across the ten genomes (no projections were available for spelt) (Table 3). This was comparable to what was observed in rice and *Ae. tauschii* across the corresponding region and higher than the gene content observed in *B. distachyon* and barley. We predicted putative NLR domains with NLR-Annotator [40] in these regions and identified between 13 to 18 NLR loci across varieties (Figure 1, Supplementary Table S1). As NLR-Annotator does not predict ORFs, we investigated whether these putative NLR loci overlapped with projected gene models from the Chinese Spring reference genome; a low proportion did so (Supplementary Table S3). Therefore, we performed a 6-frame translation of the NLR loci predicted by NLR-Annotator to determine whether there was evidence of an ORF and to determine if nearby sequences encoded BED domains. Of the 13 to 18 predicted NLR loci across varieties, we found between 4 to 10 BED-NLRs (Table 3). Overall, the number of NLR and BED-NLR loci across the *Yr* regions were higher in the wheat cultivars investigated here than in related grasses (Table 3), confirming our previous observation [18]. The functional role of these multiple BED-NLR loci remains to be determined.

Amongst the wheat varieties investigated here, Spelt PI190962 displayed the lowest number of BED-NLRs (four including Album-*Yr5a,* Table 3), whereas their number slightly expanded in Group 3 varieties (six to seven). The expansion in the BED-NLR numbers was even more pronounced in Group 1 and Group 2 varieties, ranging from eight to ten (Table 3). The BED-NLR cluster containing the *Yr7*-L744P allele in Landmark, Mace, and Stanley (Figure 2, Group 1) is located within the TraesCS2B02G488200/TraesCS2B02G488500 rearrangement. There were only two or three BED-NLRs in Group 2 and Group 3 varieties between TraesCS2B02G488200 and TraesCS2B02G488500, whereas we identified between four to seven BED-NLRs in Group 1 varieties (Figure 1). This interval did not contain NLRs in Spelt PI190962. A comparable expansion in BED-NLRs seems to have occurred in Group 2 varieties between TraesCS2B02G488700 and TraesCS2B02G489500 which contain five to six BED-NLR loci compared to only one or two in Group 1 and 3 varieties, respectively. Additionally, most of NLRs and BED-NLRs that are in synteny within a Group share high sequence similarity (Supplementary Tables S3 and S4, Supplementary Figure S3). However, outside the three main Groups, the NLRs and BED-NLRs do not share high sequence similarity; most sequence identities are below 50% (Supplementary Figure S3, Supplementary Table S2). The variation described above at the organization and architecture levels coincides with variation in the number of BED-NLR loci present within these re-arrangements and implies that this genomic region is dynamic in modern wheat varieties.

**Table 3:** Gene content with number of NLR and BED-NLR loci in *Yr* locus across chromosome scale assemblies of wheat genomes and related grasses.

	Genome	Region Size (Mb)	# Annotated Genes	# NLR Loci	# NLR Loci Overlapping with Gene Models	# BED-NLRs	# BED-NLRs Overlapping with Gene Models
	Spelt PI190962	1.9	-	13	-	4	-
Group 1	Landmark	3.6	40	18	7	9	2
	Mace	3.2	39	18	5	10	2
	Stanley	3.2	39	18	5	9	1
	SY-Mattis	3.1	41	15	5	8	1
Group 2	Lancer	2.2	32	13	4	9	2
	Julius	2.2	34	13	5	9	2
Group 3	Jagger	4.7	54	14	9	6	3
	Arina	4.6	54	14	8	6	3
	Norin61	4.5	46	17	8	7	3
	Chinese-Spring	4.5	42	14	10	6	3
Related grasses	*Ae. Tauschii* *	3.8	39	8	8	4	4
	Barley *	3.7	29	2	2	0	0
	*B. distachyon* *	0.096	22	4	4	4	4
	Rice *	0.143	50	6	6	2	2

#: Number of. * Data from the syntenic *Yr* region described in Marchal et al., 2018 [18]. DNA sequences of all NLR loci is available in File S1.

### 3.3. BED Domains from BED NLRs Share Similarities with BED Domains from Single-BED and BED-DUF-hAT Proteins

The ‘integrated decoy’ model [9] proposes that NLR-IDs recognize the pathogen either by direct binding of an effector or perception of a post-translational modification on its guardee [5,6,7,8,10]. We hypothesize that BED domains act as an integrated domain involved in effector recognition. This model predicts that integrated domains share some degree of sequence and/or structure similarity with the host target of the effector. Based on this, integrated domains might have evolved to strengthen the interaction with pathogen effectors after integration. This could lead to different outcomes; one in which the effector and integrated domain have co-evolved independently of the original host target which would result in relatively low sequence conservation between the NLR-ID and the host target [24]. A second outcome would be that the integrated domain and host target have maintained relatively high sequence similarity due to maintaining binding specificity. Hence, we hypothesized that comparing the sequences of the BED domains from BED-NLRs with the most similar BED domains from other proteins could inform on the putative plant target and the origin(s) of the integrated domain in BED-NLRs. To investigate this, we retrieved all BED-containing proteins in wheat and related Pooideae species showing an acceptable BUSCO score (see Methods and Supplementary Table S5). Across these six species, we identified a total of 151 BED containing proteins, of which 51 orresponded to BED-NLRs (Pooideae, orange group, Supplementary Table S6). The ratio of BED-NLRs to total BED-containing proteins varied between species, ranging from 11% in *B. stacei* (one in nine) to 60% in *T. dicoccoides* (12 in 20).

We carried out a neighbor-network analysis of the 151 Pooideae BED domains, with the addition of BED domains from Yr7, Cadenza-Yr5, Yr5a and Rph15 [17,18] (155 BED domains in total), to determine whether BED domains from BED-NLRs shared similarities with those from BED-containing proteins (Figure 3). We hypothesized that integrated BED domains may be under different evolutionary constraints than BED domains from the original host target(s) and thus should cluster separately. Consistent with our wheat-centric analysis [18], all the Pooideae BED domains from NLRs grouped together, into two separate clusters (44 in cluster I and 11 in cluster II) (Figure 3). This network topology is consistent with our hypothesis that BED-NLRs may have arisen from different integration events or have highly diverged from an ancient integration event. Interestingly, BED domains from a few non-NLR proteins grouped very closely with BED domains from NLRs (e.g., TraesCS2B02G488600.1 in cluster I). Although this observation is consistent with the hypothesis that both ID and effector target maintained high sequence similarity due to maintaining binding specificity, one possible explanation is that these BED domains may be truncated BED-NLRs that have accumulated premature termination codons in their coding region as we observed for *nlr_11b* in Cadenza (Supplementary Figure S4).

To address this, we further investigated seven BED domains from six non-NLR proteins which clustered most closely with BED-NLRs in clusters I (six) and II (one) (AET6Gv20991600, AET2Gv21027500, TraesCS2B02G488600, TraesCS2A02G466500, AET3Gv21007600, TRIDC6BG073250, Figure 3). All these proteins encoded only one or two BED domains and no other conserved domain. We examined the surrounding gene models to determine whether they were located in NLR-related regions (Supplementary Table S7). Five BED proteins (representing six BED domains) were located close to NLR-related regions and all share similarities with BED domains from BED-NLRs proteins (Supplementary Table S7). This suggests all these closely related non-NLR BED domains in clusters I are possibly truncated BED-NLRs (Supplementary Table S7). We extended this analysis to the other Pooideae non-NLR proteins encoding a single BED domain that were located outside the two main BED-NLR clusters, to determine whether they could be truncated BED-NLRs (Supplementary Table S9). We identified two more loci that were located less than 21 kb from an NLR-related sequence (Bradi3g14893.1 and HORVU6Hr1G010990, Supplementary Table S9), although they did not belong to the BED-NLR clusters. However, none of the best BLAST hits of Bradi3g14893.1 and HORVU6Hr1G010990 (to *B. distachyon* and barley, respectively) possessed NLR-related domains (Supplementary Table S9). It could thus be that either these proteins are located in the vicinity of NLR-related sequences by chance, or they are recent insertions of non-NLR proteins in NLR-related regions. This was consistent with the network presented in Figure 3 showing that these two proteins are more sequence divergent to BED-NLRs (Bradi3g14893.1 and HORVU6Hr1G010990).

In the previous analysis we found that all BED domains from BED-NLRs grouped together in cluster I and II (Figure 3). Additionally, all non-NLR proteins interspaced with BED-NLRs in cluster I and II showed similarities to BED-NLR sequences (Supplementary Table S7). We thus sought to determine whether the non-NLR proteins with BED domains showing slightly more sequence divergence to BED-NLRs in clusters I and II had a conserved domain organization that could inform on the possible origin of the integration of the BED domain in NLRs. We first identified all additional domains among BED-containing proteins, in additions to protein solely containing a BED domain(s) (Supplementary Tables S11 and S12). We next determined the proportion of these domains in non-NLR proteins overall and in BED-NLR clusters and carried out an enrichment analysis to identify the domains that were significantly overrepresented in BED-NLR clusters (Fisher’s exact test *p*-value < 5 × 10^−2^, Table 4, Supplementary Table S14). We excluded domains related to NLR proteins to avoid bias. We identified three domains that were significantly overrepresented in BED-NLR clusters (*p*-value < 5 × 10^−2^); hAT family C-terminal dimerization region (hAT, PF05699), Domain of unknown function (DUF) 4413 found in transposase-like proteins (DUF4413, PF14372) and single-BED proteins (Table 4). These domains were organized in the following protein architectures: single-BED, BED-DUF4413(-Dimer_Tnp_hAT) (Supplementary Table S12). 

We performed a similar analysis in other plant species containing BED-NLRs to compare the results from Pooideae. We investigated an additional 84 plant proteomes and selected 63 for the BED domain analysis based on their BUSCO scores (see Methods, Supplementary Table S5). Fourteen of the 63 proteomes contained BED-NLRs and we separated the proteomes in four groups of phylogenetically related species: Ehrhartoideae (brown group), Panicoideae (yellow group), Fabideae + Eucalyptus Grandis (blue group), and Malpighiales (green group) (Supplementary Table S6). We carried out a similar neighbor-network analysis on their BED domains as in Pooideae (Supplementary Figures S5–S8, Supplementary Tables S8, S10, and S13). We observed a similar topology to that of the Pooideae in the four studied groups with most of the BED-NLRs clustering in one or two groups (e.g., in the Malphigales all BED-NLRs grouped in one cluster, as shown in Supplementary Figures S5–S8). This was true even in groups with low number of BED-NLRs (e.g., Panicoideae with 13/14 BED-NLRs in cluster I and the remaining BED-NLR in cluster II). We analyzed the flanking regions of the 20 BED domains from non-NLR proteins interspaced with BED-NLRs and found that 13 single-BED coding proteins were located in NLR-related regions based on the same criteria used for Pooideae (Supplementary Table S8). Additionally, all 13 single-BED proteins located in NLR-related regions were also shared sequence similarities with BED-NLRs in the corresponding species (BLAST analysis, Supplementary Table S8). Most of these putative truncated BED-NLRs were identified in *Eucalyptus grandis* and *Populus trichocarpa* (Supplementary Table S8). There were two BED-containing proteins carrying additional domains that were interspaced with BED-NLRs, suggesting that BED domains from BED-NLRs share more similarities to BED domains from non-NLR proteins in these groups (Supplementary Table S8). We identified seven more single-BED coding proteins located in NLR-related regions when exploring the regions surrounding all single-BED coding proteins with NLR-Annotator (Supplementary Table S10). However, BLASTp analyses showed that one of them, Eucgr.C00524.1, shared more similarities with a BED domain from BED-DUF4413-hAT protein and six other single-BED proteins did not have a relevant BLAST hit covering at least 90% of the query sequence (Supplementary Table S10). Apart from their genomic location within 21 kb from NLR-related region, there was thus no evidence for these single-BED proteins to be related to NLRs.

**Table 4:** Conserved domain enrichment analysis in BED-containing proteins whose BED domains share similarities with BED domains from BED-NLRs in the Pooideae (Fisher’s exact test with alternative hypothesis = greater).

Domain	Pfam	In Cluster with BED-NLRs	In all BED Containing Proteins	Fisher Exact Test (*p*-Value, Alternative Hypothesis = Greater)
Dimer Tnp hAT	PF05699	8	14	6.18 × 10^−3^ *
DUF4413	PF14372	9	14	1.04 × 10^−3^ *
single-BED ^1^	PF02892	22	49	1.56 × 10^−4^ *
All domains in BED-containing proteins (minus NLR-related domains)	-	39	121	-

^1^ BED-only coding genes located in NLR-related regions were removed. * *p*-values < 5 × 10^−2^

We then investigated whether the overrepresented protein architectures found in BED-NLR clusters in the Pooideae were also identified in these three groups (Table S14). We found the following domains significantly enriched in BED-NLRs clusters: single-BED proteins (four out of five groups), DUF4413 (2/5 groups), DUF659 (one in five groups) and hAT (one in five groups). Additionally, Calmodulin binding domains were overrepresented in BED-NLR clusters in the Fabideae exclusively (Supplementary Table S14). Note that we did not find any positively enriched domains in the Malphigales given that only one non-NLR protein clustered close to BED domains from BED-NLRs. This may suggest that the BED domains from BED-NLRs have diverged more from BED domains from non-NLR proteins in this group compared to the others. There was no single protein architecture that was enriched in BED-NLR clusters in all studied groups, although the following protein architectures were enriched in some: single-BED and BED-DUF4413/DU659(-hAT) (Supplementary Table S13). The identification of common protein domains in the non-NLR proteins clustering with BED-NLRs may inform on the origin of the integration. Based solely on sequence similarity, our results suggest multiple possible origins for the integration of the BED domain in NLRs depending on the species studied. This knowledge will help prioritize targets for future experimental work to confirm this hypothesis, determine the functional role of these non-NLR BED proteins and their relationship, if any, with BED-NLR-mediated resistance.

### 3.4. Functional BED-NLRs Carry a Nuclear Localisation Signal

Nuclear localization signals (NLS) are present in rice BED-NLRs *Xa1* and the *Xo1* [16]. Given that *Xa1* was distant, but still phylogenetically related to Yr7 and Yr5a [16,18], we looked for the presence of NLS in the BED-NLRs. We identified an NLS within 40 amino-acids downstream/upstream of the BED domain in a 30 out of 74 BED-NLRs in monocot species, including the functional Album-Yr5a and Cadenza-Yr7 proteins (33 amino-acids downstream of the BED domain) (Supplementary Figure S5). Interestingly, the recently characterized Rph15 resistance protein also carries an NLS in the vicinity of the BED domain and its location and sequence are similar to that of Xa1. The predicted NLS were positionally conserved across similar BED-NLR proteins, including BED-NLRs carrying two consecutive BED domains (Supplementary Figure S5). Therefore, the presence of the putative NLSs, albeit different in sequence and position, is a common feature of the five BED-NLR immune receptors that have been functionally characterized in rice and wheat (Supplementary Figure S5, [16,17]).

We tested if the Yr7 NLS is functional and necessary to localize the Yr7 protein into the nucleus. We generated three truncations of the Yr7 protein tagged with YFP to determine their cellular localization (Figure 4). All three truncated proteins include the BED domain, but differed in the size of the region preceding the NB-ARC domain which includes the putative NLS. The Yr7-AA201 truncation which does not carry the NLS showed a nucleo-cytoplasmic localization, similar to that of YFP alone (Figure 4). However, Yr7-AA242 and Yr7-AA308, both carrying the predicted NLS, strictly localized in the nucleus (Figure 4). To confirm that the NLS was responsible for this localization, we generated deletion mutant in Yr7-AA242 and Yr7-AA308 lacking the seven amino acids of the putative NLS. Both truncations displayed nucleo-cytoplasmic localization similar to that of YFP alone (Figure 4). Therefore, we conclude that the predicted NLS is functional in *N. benthamiana* in the context of these truncations. It remains to be determined whether this localization is maintained in full-length Yr7 and its importance for resistance against the yellow rust pathogen in wheat.

Given the presence of a NLS close to the BED domain in functional BED-NLRs, we asked whether there was a correlation between the presence of an NLS in BED-NLRs and non-NLR proteins across the cluster we defined in the neighbor-networks [Figures S5–S7]. We did not find a clear relationship as not all BED domains from non-NLR proteins that grouped with BED-NLRs carried an NLS and *vice-versa*. Additionally, where present, the NLS was localized further away from the BED domain in dicots compared to monocots (Table S15). Indeed, 97% (30/31) of BED-NLRs with an NLS in monocots had their NLS within 40 amino-acid from the BED domain, compared to only 1 in 8 in dicots. This feature is not unique to BED-NLRs as we also observed this in non-NLR BED proteins (83% (144/173) of monocots BED-containing proteins carry an NLS within 40 amino-acid from the BED domain versus 49% (50/103) of dicots). Thus, the NLS is preferentially localized in close-proximity to the BED domain in both non-NLR and BED-NLRs in monocots, whereas this pattern is observed less often in dicots. Based on these observations, it is unlikely that the NLS is part of the integration of the BED domains in dicot NLRs. Indeed, there might be other conserved domains between BED and NLS in non-NLR proteins and these domains are not present in BED-NLRs. For monocots, although the NLS can flank the BED domain of BED-NLRs (e.g., *Xa1* and Rph15), it can also be located slightly further away from the BED domain, as in Yr7 and Yr5 (33 amino-acids). Therefore, it remains unclear whether the NLS is part of the BED domain integration in NLRs. Despite this, given that the five characterized BED-NLRs contain an NLS and that this NLS is functional in the Yr7 truncation, and N-ter *Xo1* [16], we hypothesize that this feature is important for BED-NLR function.

### 3.5. Yr7 and Its Variants do not Trigger Cell-death upon Overexpression in N. Benthamiana

Unlike well-characterized NLR-IDs, we did not identify a potential paired canonical NLR in the vicinity of *Yr7*. We thus hypothesized that either *Yr7* could act as a potential singleton NLR and is able to both detect pathogens and trigger cell-death. Several characterized singletons are able to trigger cell death in heterologous plant systems independently of the presence of the effector. These include *Mla10*, *Sr50, RPP13, RPS5, ZAR1,* and *L6* (reviewed in Adachi et al., 2019 [65]). Alternatively, *Yr7* might rely on a helper to signal, which is not located in close-proximity in the genome (e.g., NRC, ADR1 and NRG1 networks [31,32,33,34,35]). We thus tested whether *Yr7* was able to trigger cell death in *N. benthamiana* to determine whether it shares this characteristic of potential singleton NLRs. We did not detect cell-death upon overexpression of Yr7 nor any of its variants, including different N-terminus truncations (Figure S10). To determine whether Yr7 was able to signal in *N. benthamiana*, we generated a D646V mutant in its ‘MHD’ motif. Such mutations enable certain ‘helper’ NLRs to trigger cell-death in *N. benthamiana* independently of the presence of the effector [59,60,61]. Again, we did not detect a hypersensitive response upon overexpression of the Yr7-D646V mutant, both with or without co-infiltration of the tomato bushy stunt virus suppressor of posttranscriptional gene silencing P19 (Supplementary Figure S10). We confirmed expression of the constructs and accumulation of the protein through western blots (Supplementary Figure S10). Given that we did not observe any cell-death phenotype linked to Yr7, it is unclear whether Yr7 does not signal in *N. benthamiana* because it relies on an external helper not present in this system or if *N. benthamiana* lacks the downstream components necessary for Yr7-mediated signaling.

## 4. Discussion

### 4.1. There is no Evidence for a NLR-partner in Yr7/Yr5 Locus: could Truncated nlr_11 be Functionaly Relevant?

We explored multiple wheat genome assemblies and identified two closely related *Yr7* sequences, the functional *Yr7* from Cadenza, and a single amino acid substitution allele *Yr7*-L744P in Landmark, Mace and Stanley (Table 2, Figure 1 and Figure 2). Cadenza shared similar genomic organization with Landmark and Mace and in all three varieties there was a predicted BED-NLR locus, *nlr_11*, located ~4.5 kb distal and in a tail to head orientation (Figure 2). Using RNA-Seq data, we show that the Cadenza *nlr_11* allele (*nlr_11b*) encodes a truncated BED-NLR (Supplementary File S2).

In first instance, the truncated nature of *nlr_11* would argue against its functional relevance. However, we previously showed that a truncated version of *Yr5* in the cultivar Spaldings Prolific (*Yr5b*) is functional and able to confer resistance to the wheat yellow rust pathogen [18]. Yr5b carries the complete N-terminus, BED and NB-ARC domains, unlike NLR_11 which encodes only the N-terminus and a partial BED domain (Supplementary Figure S4 and Supplementary File S2). We therefore examined for the presence of coiled-coil domains in the N-terminus. Different prediction programs provide conflicting results on the presence of a CC domain in the N-terminus upstream of the BED domain in Yr7, nlr_11, and Yr5a/b [17,18]. However, structure prediction programs such as Phyre2 [66] predict a CC-like structure upstream the BED domain of Yr7 and Yr5a/b sharing high structural homology (>99.7%) with CC domains from ZAR1 [67,68] and Mla10 [69]. Similar results were also obtained for NLR_11, suggesting that this truncated BED-NLR protein includes a CC domain. Truncated NLRs have a role in plant disease resistance, for example, truncated TIR and TIR-NB proteins are required for cell-death signaling upon pathogen recognition [70,71,72]. However, although CC domains from several CNLs are able to trigger cell-death independently of the presence of the pathogen [62,69], there are no reports of a truncated functional CCs involved in plant disease resistance to our knowledge. The closest example would be the RPW8 genes that encode short proteins encoding a particular CC domain and confer broad spectrum resistance against powdery mildew in Arabidopsis [73]. Although the CC domain of RPW8 share similarities with CC domains derived from a particular class of CNLs that includes ADR1 and NRG1 (RWP8-like CC-NLRs, [32,33,34,35]), there is no known link between the mode of action of these two RPW8-containing proteins [74]. Alternatively, *nlr_11* could be a degenerated BED-NLR. It therefore remains to be determined if the NLR_11 truncation plays a role in *Yr7*-mediated resistance.

### 4.2. Mode of Action of BED-NLRs may be Different that of Known NLR-IDs

Here, we identified two instances of functional BED-NLR loci (*Yr7* and *Yr5a*) lacking a NLR partner in the cultivar carrying the functional alleles. We thus explored whether this was consistent with other BED-NLRs. A long-range assembly of Carolina Gold Select rice variety was recently released and this accession carries *Xo1*, which encode a BED-NLR [75]. Using this assembly and its available NLR annotation, we explored the genomic region surrounding *Xo1* and noticed that the closest NLRs were located 50 kb distal and ~230 kb proximal to *Xo1*, reminiscent of the *Yr5a* locus in spelt (Figure 1 and Supplementary Figure S11). The absence of partner NLRs could thus be a feature of BED-NLRs and suggests a mode of action different from characterized NLR-IDs.

Interestingly, Yr7 and the BED-NLRs included in this study do not carry the predicted MADAxVSFxVxKLxxLLxxEx motif that covers most of the alpha1 helix of ZAR1, which is essential for the activation of the resistosome [68,76,77]. Given that this motif was primarily found in singletons and helper NLRs [77], its absence in BED-NLRs may suggest that they could rely on a NLR helper for cell-death signaling upon pathogen recognition. Resolving the structure of the N-terminus of Yr7 and other functional BED-NLRs and comparing it to characterized CC folds from singletons will help address this hypothesis.

### 4.3. N. benthamiana May not Be a Suitable Heterologous System to Study Signalling Mediated by BED-NLRs

Based on known NLR mechanisms, the lack of a canonical NLR partner for *Yr7* (and *Yr5*) may suggest two hypotheses: Yr7 could be a singleton that is able to both detect and signal the presence of the pathogen (e.g., *Mla10* [62] and *Sr50* [78] in cereals), or Yr7 may require an additional component that is not genetically linked (e.g., NRC, ADR1 and NRG1 networks [31,32,33,34,35]). However, we did not observe cell-death in *N. benthamiana* transiently expressing full-length Yr7, its derived truncations or an MHD variant (Supplementary Figure S10). It is thus unclear whether *N. benthamiana* is a relevant heterologous expression system for Yr7 and BED-NLRs in general. Indeed, we did not find any BED-NLRs when we explored *N. benthamiana* proteome [79]. Thus, *N. benthamiana* may lack the components mediating BED-NLR signaling. An alternative would be to use luciferase assays in wheat protoplasts transiently overexpressing Yr7 and its variants to determine their ability to trigger cell-death directly in the host plant. Indeed, this system successfully recapitulates cell-death upon the co-expression of AvrSr50 and Sr50 [80] and could thus be used to investigate *Yr7*-mediated signaling. 

### 4.4. BED Domains from BED-NLRs Share Similarities with BED Domains from Single-BED and BED-DUF4413/659-(hAT) Proteins

According to the ‘integrated decoy’ model, NLR-IDs arose from the integration of an effector target domain (e.g., BED domain) in the canonical NLR structure [9]. Therefore, we hypothesized that comparing the sequences of the BED domains from BED-NLRs with the most similar BED domains from other host proteins could inform on the putative host targets of pathogen effectors and the origin(s) of the integration event into the BED-NLRs. In Pooideae, and other plant families, we found that most BED domains from NLR proteins were closely clustered (Figure 3 and Supplementary Figures S5–S8). Few if any, BED domains from bona fide non-NLRs proteins clustered as closely with BED-NLRs. This suggests that BED domains from NLR proteins are under distinct selection pressure to BED domains from non-NLR proteins and hence it will be difficult to predict putative host targets based on sequence similarity alone. 

With this knowledge, we clustered BED domains empirically, focusing on BED domains from non-NLRs proteins located in the closest groups to BED-NLRs. We assumed that this degree of variation would be similar to that between an ID and the corresponding effector target. We conducted a similar analysis on the WRKY domains from non-NLR and NLR proteins in Arabidopsis, as *RRS1*, an NLR-WRKY [30], and *PopP2*, a *Ralstonia solanacearum* effector, are a better characterized system than BED-NLRs (Supplementary File S5). We observed that half of the non-NLR WRKYs that are acetylated by PopP2 and thus potential effector targets cluster near NLR-WRKYs (Supplementary File S5). As they would have thus been included in the neighbor-network analysis, we argue that this approach can help prioritize targets for further functional validation of potential effector targets and determine whether they play a role in corresponding NLR-ID-mediated resistance. Using this empirical clustering on BED domains, we identified five domains that were significantly enriched in BED-NLR clusters, although no domain was enriched in all five studied groups (Figure 3 and Supplementary Figures S4–S7, Table 4 and Supplementary Table S13). Despite this, we identified single-BED and BED-DUF4413/659(-hAT) protein architectures in BED-NLR clusters from all five networks (Supplementary Tables S11 and S12). Given that different protein architectures were enriched in BED-NLR clusters from different species, it would suggest that the integration of BED domains in NLRs have occurred multiple times in plant evolution. This hypothesis will need to be addressed in future experimental work. One important caveat of this approach is that it does not consider structural homology, which can be conserved between divergent sequences (e.g., Pikm-HMA and Pikp-HMA [81]). However, the only available BED domain structure is derived from the human protein ZBED2 [82], making modelling of plant BED domains in a high-throughput manner difficult. 

### 4.5. Nuclear Localization Signal is a Feature of BED-NLRs that Confer Resistance to Plant Pathogens

Identifying and validating nuclear localization signals is important given that nuclear localization of certain NLRs is required for resistance, as is the case for barley Mla10 and *Arabidopsis* RPS4 [83,84]. In this study, we identified NLS in 28 out of 67 BED-NLR from bread wheat and related species; this includes *Xa1*, *Xo1*, Yr7, Yr5a/b, and the recently cloned barley BED-NLR Rph15 (Supplementary Figure S5). Note that the N-terminus of Xo1 localizes in the nucleus [16]. The position of the NLS varied, however. In the case of *Xa1*, *Xo1* other rice BED-NLRs, Rph15, and BED-NLRs from wheat, wild emmer and *Ae. tauschii* carrying a BED domain related to BED-II class [18] the NLS flanked the BED domain on its 5’ end. However, in Yr7, *Yr5,* and other BED domains more related to the BED-I type, the NLS was located ~30–35 amino acids 3’ of the BED domain (Supplementary Figure S5). Therefore, not all BED-NLRs contain an NLS, and where present, the position within the protein varies. We provide experimental support, in *N. benthamiana,* for the predicted NLS in a truncated version of the Yr7 protein (Figure 4). It remains to be tested, however, whether the NLS is required for the expression of the *Yr7*-mediated resistance in wheat. Although NLS were not found in all BED-NLRs, the five BED-NLRs characterized to confer resistance to pathogens carry one in the vicinity of the BED domain (Supplementary Figure S9).

We performed a series of comparative genomics, bioinformatics, and functional experiments to further elucidate the role of BED domains in BED-NLR mediated resistance. These findings may have implications regarding the origins of the integrations of the BED domain in BED-NLRs and will help prioritize host targets to decipher their mode of action. Further investigation of the role of single-BED and BED-DUF4413/659-(hAT) proteins is warranted, as is the functional characterization of truncated BED-NLRs and those closely related to the functional cloned genes. Understanding the importance of protein structural homology between BED-NLRs and putative host targets, as opposed to sequence homology, also remains a major limitation in our understanding. The fact that we did not identify paired NLRs in the genomic context of independent functional BED-NLRs suggests that their mode of action might differ to currently characterized paired NLR-IDs. Likewise, the validation of the NLS also raises further questions: Is the NLS a requirement for the function of BED-NLRs and could it be used as a criterion to differentiate between functional and non-functional BED-NLRs? Or would BED-NLRs have different functions depending on the presence/absence and the position of an NLS? These questions will need to be addressed to further understand the role of BED domains in BED-NLR mediated resistance. 

## Figures and Tables

**Table 1:** Summary of the ten chromosome-scale wheat assemblies used in the present study (http://www.10wheatgenomes.com/progress/) [36].

Cultivar	Name Used in This Study	Region of Origin
Julius	Julius	Germany
Jagger	Jagger	USA
Norin61	Norin	Japan
CDC Landmark	Landmark	Canada
CDC Stanley	Stanley	Canada
Arina*LrFor*	Arina	Switzerland
Mace	Mace	Australia
LongReach Lancer	Lancer	Australia
SY-Mattis	SY-Mattis	France
Spelt PI190962	Spelt PI190962	Europe

## Supplementary Materials

The following are available online at https://www.mdpi.com/2073-4425/11/12/1406/s1, **Figure S1:** Schematics representing the Yr7 and Yr5 alleles and best hits in sequenced wheat cultivars. BED domain is shown in red, NB-ARC in blue and LRRs in green (light green for manually annotated LRR and dark green for LRR motifs derived from NLR-Annotator [40]). Unique amino-acid polymorphisms across different haplotypes are depicted in light blue and shared polymorphism across at least two haplotypes in orange. Red stars show premature termination codons. Known functional genes/alleles conferring resistance to the wheat yellow rust pathogen are highlighted in pink. The black arrow points to the position of the single SNP identified between Cadenza-Yr7 and Landmark/Mace/Stanley-Yr7 (Yr-L744). **Figure S2:** Heatmap of the best BLAST hits of RefSeqv1.1 gene models located in the chromosome 2B *Yr* region across wheat varieties. Hits were reported if they covered at least 90% of the query. Best BLAST hits were defined based on bitscore, evalue and percentage identity (see Methods). We used a gradient from dark to light blue to reflect the percentage identity between the RefSeqv1.1 gene model and its corresponding hits (Table S1), with dark blue representing perfect hits and light blue hits that are ~80% identical. Genes in red text contain canonical domains from NLRs. Stars depict genes showing hits in all varieties. **Figure S3:** Heatmap representing the percentage of sequence similarity between NLRs located in the *Yr* region. We used a gradient from dark green to light green to represent sequences sharing from 50 to 90% sequence identity and a gradient from light blue to mild blue to show sequences sharing from 90 to 99.9% identity. Dark blue represents sequences sharing 100% identity. Sequences sharing less than 50% sequence identity are showed in white. The data used to generate this heatmap are presented in Table S2. **Figure S4:** Gene structure annotation of Cadenza *nlr_11b*. RNA-seq reads from Cadenza mapped to the Cadenza genome assembly (grey) with range of coverage shown in black. We determined the corresponding exon/intron structure based on the RNA-seq reads and identified potential BED and NB-ARC domains when translating the gDNA sequence in the six frames. Premature termination codons are shown in red in the corresponding exons. **Figure S5:** Neighbour-network of BED domains from non-NLR and NLR proteins in the Ehrhartoideae. The network was generated as for Figure 3 and caption, shapes and colours reflect the same features as in Figure 3. **Figure S6:** Neighbour-network of BED domains from non-NLR and NLR proteins in the Panicoideae. The network was generated as for Figure 3 and caption, shapes and colours reflect the same features as in Figure 3. **Figure S7:** Neighbour-network of BED domains from non-NLR and NLR proteins in the Fabideae and *E. grandis*. The network was generated as for Figure 3 and caption, shapes and colours reflect the same features as in Figure 3. **Figure S8:** Neighbour-network of BED domains from non-NLR and NLR proteins in the Malpighiales. The network was generated as for Figure 3 and caption, shapes and colours reflect the same features as in Figure 3. **Figure S9:** Alignment of the region surrounding the BED domains (40 amino-acid up- and downstream) of Pooideae and Ehrhartoideae NLRs carrying an NLS. Alignment was generated with MAFFT [53] v7.305 using default parameters and visualized with Jalview [55] v2.10. The gradient of purple depicts the degree of conservation at a given position, which is shown in the graph underneath each alignment. The red boxes mark the boundaries of the BED domains and the pink boxes those of the predicted NLS. NLR carrying two BED domains have their names in red. **Figure S10:** Cell-death assays in *N. benthamiana* upon overexpression of Yr7 protein and its variants. Top to bottom of each pane; schematic representing the Yr7 variants analysed with the corresponding immunoblots and picture of the infiltrated *N. benthamiana* leaved 5–7 days post infiltration (dpi). Pikp-2 was used as a negative control for cell-death and Mla10 as a positive control. Infiltration areas are shown in dashed red circle. Bottom pictures were taken with an UV light and were swapped to reflect the same orientation as the corresponding picture in normal light. A, Transient expression optimization of Yr7 full-length, including 6xHA tag at C-terminus, at different OD_600_ and different time points post-infiltration. B, Transient expression of the Yr7-D646V variant with and without co-infiltration with an *A. tumefaciens* transformed with the P19 silencing suppressor [64]. C, Transient expression of different truncated variants of the Yr7 protein with YFP tag at the C-terminus (yellow). The samples at 2 dpi of the second western blot show possible degradation of the truncation with a band whose size is very close to that of YFP alone. **Figure S11:** Schematic of the *Xo1* region in the rice cultivar Carolina Golden Select. The assembly and corresponding NLR annotation and *Xo1* sequence were obtained from Read et al., 2020 [75]. Triangles show the position of the NLRs with black triangles depicting canonical NLR loci and red triangles BED-NLR loci based on CD-search prediction from Read et al., 2020 [75]. **Table S1:** Percentage identity values used to generate the heatmap on Figure 1**.** Only BLAST hits covering at least 90% of the query sequence were retained. Hits were then sorted according to their bitscore and evalue to select the best hit on chromosome 2B within the *Yr* region. Green highlight indicates genes that showed hits across all genomes. It is important to note that this table only reports the best hit within the Yr region of each genome; better hits may be located elsewhere. **Table S2:** Percentage identity matrix showing the degree of similarity between NLRs located in the Yr region across eleven wheat genomes. We retrieved all NLR loci predicted by NLR-Annotator [40] and used Clustal Omega (https://www.ebi.ac.uk/Tools/msa/clustalo/ [41]) to produce a multiple sequences alignment and the corresponding percentage identity matrix to reflect the degree of conservation between putative NLR loci located in the Yr region across the eleven wheat genomes studied here. **Table S3:** NLR-loci identified in multiple wheat varieties with NLR-Annotator. Where possible, the gene model overlapping the NLR-Annotator locus is indicated and the corresponding protein coding sequence was used to predict the presence of conserved domains. When no projected gene model was overlapping with a predicted NLR locus, we performed a six-frame translation with EMBOSS Transeq (https://www.ebi.ac.uk/Tools/st/emboss_transeq/) to identify potential conserved domains. NLR loci highlighted in red were not included in Figure 1 for the reasons highlighted in the comment section. **Table S4**: List of the potential NLR alleles in the *Yr* region across the multiple wheat cultivars. Data is based on BLAST results selecting for 100% identity across 100% of the query sequence. **Table S5:** Downloaded proteomes for the study and associated BUSCO scores based on the Viridiplantae and Embryophyta datasets. We retained proteomes that obtained a percentage completeness greater than 85% for both Viridiplantae and Embryophyta datasets from Embryophyta plants and greater than 85% for the Virdiplantae dataset for non-Embryphyta plants. Green colour identifies selected proteomes and red non-selected ones based on these criteria. The presence of BED-containing proteins and BED-NLRs is indicated in the last two columns (Y for yes and N for no). **Table S6**: BED-NLR and non-NLR BED proteins in proteomes from species containing BED-NLRs. **Table S7**: Domain organization of proteins surrounding BED-proteins whose BED domain closely clusters with BED domains from BED-NLR in Pooideae (orange group). Conserved domain identification was carried out with HMMER [42] v.3.1 as described in the methods section. We report the protein size and its best BLASTp hit in the NCBI database (https://blast.ncbi.nlm.nih.gov/) with the corresponding percentage identity between the two sequences and the conserved domains present in the best BLAST hit. We only report hits for which >90% of the query sequence length was covered by the alignment. **Table S8**: Domain organization of proteins surrounding BED-proteins whose BED domain closely clusters with BED domains from BED-NLR in Ehrhartoideae (brown group), Panicoideae (yellow group), Fabideae (blue group) and Malpighiales (green group). Conserved domain identification was carried out with HMMER [42] v.3.1 as described in the methods section. We report the protein size and its best BLASTp hit in the NCBI database (https://blast.ncbi.nlm.nih.gov/) with the corresponding percentage identity between the two sequences and the conserved domains present in the best BLAST hit. We only report hits for which >90% of the query sequence length was covered by the alignment. **Table S9:** Genomic regions harboring NLR-related motifs in sequences flanking genes encoding BED domain(s) (±21 kb up- and downstream each BED and BED-BED encoding gene) in Pooideae (orange group). In orange, loci that are interspaced with BED-NLR in the neighbour-net analysis and that were located in NLR-related region based on Table S7. The data represents the output from NLR-Annotator [40]. We report the protein size and its best BLASTp hit in the NCBI database (https://blast.ncbi.nlm.nih.gov/) with the corresponding percentage identity between the two sequences and the conserved domains present in the best BLAST hit. We only report hits for which >90% of the query sequence length was covered by the alignment. **Table S10:** Genomic regions harboring NLR-related motifs in sequences flanking genes encoding BED domain(s) (±1 kb up- and downstream each BED and BED-BED encoding gene) in Ehrhartoideae (brown group), Panicoideae (yellow group), Fabideae (blue group) and Malpighiales (green group). In orange, loci that are interspaced with BED-NLR in the neighbour-net analysis and that were located in NLR-related region based on Table S8. The data represents the output from NLR-Annotator [40]. We report the protein size and its best BLASTp hit in the NCBI database (https://blast.ncbi.nlm.nih.gov/) with the corresponding percentage identity between the two sequences and the conserved domains present in the best BLAST hit. We only report hits for which >90% of the query sequence length was covered by the alignment. **Table S11:** Count of additional domains in BED-containing proteins in the analysed proteomes. **Table S12:** BED-containing proteins whose BED domains cluster with those of BED-NLRs in the neighbour-network analysis and corresponding additional domains in the Pooideae (orange group). Background colour represents the group defined in Table S6. The domain composition of the BED-containing proteins is indicated in the “Domains” column, the presence of Nuclear Localization Signal in the “NLS” column (#N/A: no NLS identified), and whether a particular BED-only coding protein is located in a NLR-related region in the last column. **Table S13:** BED-containing proteins whose BED domains cluster with those of BED-NLRs in the neighbour-network analysis and corresponding additional domains in Ehrhartoideae (brown group), Panicoideae (yellow group), Fabideae (blue group) and Malpighiales (green group). Background colour represents the groups defined in Table S6. The domain composition of the BED-containing proteins is indicated in the “Domains” column, the presence of Nuclear Localization Signal in the “NLS” column (#N/A: no NLS identified), and whether a particular BED-only coding protein is located in a NLR-related region in the last column. **Table S14:** Enrichment analysis of additional domains found in BED-containing proteins whose BED domains cluster with those of BED-NLRs in the neighbour-network analysis in Pooideae (orange group), Ehrhartoideae (brown group), Panicoideae (yellow group), Fabideae (blue group) and Malpighiales (green group)**.** Fisher’s exact test with alternative hypothesis = greater was performed for each group (*p*-values < 0.05 are shown in green). **Table S15:** Identification of Nuclear Localization Signals in BED-containing proteins from species with BED-NLRs. Proteins highlighted in red possess more than one NLS. The table indicates whether the protein is a BED-NLRs (BED-NLR column) and if not, whether its BED domain clustered with BED domains from BED-NLRs (in clade column). Distances between the NLS and BED domains are reported in the corresponding column and the last column shows whether the NLS is located within 40 amino-acid from the BED domain. **Table S16:** Primers used to generate the Yr7 truncation in the cellular localization experiments and HR assays in *N. benthamiana*. Primers highlighted in orange were used on the codon-optimized version of Yr7 cDNA. **Table S17:** List of the 10 + Wheat Genome Project authors. **File S1:** DNA sequences of the NLRs identified in the *Yr* region in the eleven chromosome scale assemblies of wheat. These potential NLR loci were predicted with the NLR-Annotator program [40] and do not correspond to putative ORFs. **File S2:** Sequence analysis of *nlr_11* in Cadenza (*nlr_11b*) and Landmark/Mace (*nlr_11a*), and *nlr_18* in Stanley. **File S3:** Amino-acid sequences of the BED domains used to generate Figure 3 and Figures S4–S7. BED domain boundaries were defined according to the Pfam model (PF02892) and the HMMER output (see Methods). **File S4:** GenBank files describing the plasmids generated for this study. **File S5:** Neighbour-network of WRKY domains from non-NLR and NLR proteins in *Arabidopsis thaliana*. The network was generated the same way as for the BED domain networks (Figure 3 and Figures S5–S8). Bootstrap values are shown in gold. WRKY-NLRs are depicted in red, whereas non-NLR WRKY proteins are depicted in green (positive for acetylation by PopP2 [5,6]), orange (interaction signal with PopP2 was slightly lower) and blue (negative for acetylation by PopP2 [5,6]). Non-NLR WRKY proteins which were not tested for acetylation are shown in black.

## Abbreviations

BED	BED-type zinc finger domain named after the Drosophila proteins BEAF and DREF
CC	Coiled-coil
DUF	Domain of unknown function
Dimer_Tnp_hAT	hAT family C-terminal dimerization region
NLR	Nucleotide-binding Leucine-rich repeat
NLR-ID	NLR with integrated domain
NLS	Nuclear localization signal
RPW8	Resistance to powdery mildew 8
TIR	Toll and Interleukin-1 Receptor
